# Thrombocytopenia in adult patients with sepsis: incidence, risk factors, and its association with clinical outcome

**DOI:** 10.1186/2052-0492-1-9

**Published:** 2013-12-30

**Authors:** Chakradhar Venkata, Rahul Kashyap, J Christopher Farmer, Bekele Afessa

**Affiliations:** Department of Critical Care Medicine, Mayo Clinic Health System, 1025 Marsh Street, Mankato, MN 56001 USA; Division of Pulmonary and Critical Care Medicine, Department of Internal Medicine, Mayo Clinic, Rochester, MN USA; Department of Critical Care Medicine, Mayo Clinic, Scottsdale, AZ USA

**Keywords:** Thrombocytopenia, Sepsis, Septic shock, Intensive care unit, Prognosis, Mortality

## Abstract

**Background:**

Sepsis is a major risk factor for the development of thrombocytopenia, but few studies have specifically evaluated prognostic importance of thrombocytopenia in patients with sepsis. We investigated the incidence, risk factors, and prognostic importance of thrombocytopenia in adult patients admitted to the intensive care unit (ICU) with sepsis.

**Methods:**

A retrospective analysis of patients admitted with severe sepsis/septic shock from December 2007 to January 2009 to a 24-bed medical ICU was done.

**Results:**

A total of 304 patients were included in the study. The patients' mean (±SD) age was 68.8 (±15.8) years. The majority (93.7%) had septic shock, and pneumonia was the most common infection (38.8%). Thrombocytopenia developed in 145 patients (47.6%): 77 (25.3%) at ICU admission and 68 (22.3%) during their hospital course. The median (IQR) duration of thrombocytopenia was 4.4 (1.9–6.9) days. Patients who developed thrombocytopenia had more episodes of major bleeding (14.4% vs. 3.7%, *P* < 0.01) and received more transfusions. Patients with thrombocytopenia had a higher incidence of acute kidney injury (44.1% vs. 29.5%, *P* < 0.01), prolonged vasopressor support (median (IQR): 37 (17–76) vs. 23 (13–46) h, *P* < 0.01), and longer ICU stay (median (IQR): 3.1 (1.6–7.8) vs. 2.1 (1.2–4.4) days, *P* < 0.01). The 28-day mortality was similar between patients with and without thrombocytopenia (32.4% vs. 24.5%, *P* = 0.12). However, while 15 of 86 patients (17.4%) who resolved their thrombocytopenia died, 32 of 59 patients (54.2%) whose thrombocytopenia did not resolve died (*P* < 0.01). The association between non-resolution of thrombocytopenia and mortality remained significant after adjusting for age, APACHE III score and compliance with a sepsis resuscitation bundle (*P* < 0.01).

**Conclusions:**

Thrombocytopenia is common in patients who are admitted to the ICU with severe sepsis and septic shock. Patients with thrombocytopenia had more episodes of major bleeding, increased incidence of acute kidney injury, and prolonged ICU stay. Non-resolution of thrombocytopenia, but not thrombocytopenia itself, was associated with increased 28-day mortality.

## Background

Thrombocytopenia (platelet count < 150,000/μl) is common in critically ill patients, with an estimated incidence of 20%–40% at some point during the intensive care unit (ICU) stay [1]. Thrombocytopenia is recognized as an independent risk factor for mortality in ICU patients [2, 3]. Both the nadir platelet count and a large drop in platelet count predict a poor outcome in adult ICU patients [3]. Prolonged thrombocytopenia and absence of relative increase in the platelet count were also associated with a greater risk of mortality [4]. Many studies tried to identify consequential risk factors for the development of thrombocytopenia in the ICU. Sepsis was found to be the most common risk factor in several studies [2, 5–7]. Increased severity of illness (as suggested by high Acute Physiology, Age, and Chronic Health Evaluation (APACHE) II and Sequential Organ Failure Assessment (SOFA) scores) [2, 8] and drugs (heparin, beta lactam antibiotics, and vancomycin) [5, 6] were also suggested to be risk factors for thrombocytopenia; however, these findings have not been consistent among various studies.

To date, most studies focus on the incidence and risk factors for the development of thrombocytopenia, as well as its association with clinical outcomes in general ICU populations. The data are limited about incidence of thrombocytopenia and its association with clinical outcomes in patients with severe sepsis and septic shock. There are also limited data available about the incidence of secondary consumptive thrombocytopenia, like disseminated intravascular coagulation (DIC) and thrombotic thrombocytopenic purpura (TTP) in patients with sepsis. The incidence of DIC in severe sepsis/septic shock patients is estimated from randomized control trial (RCT) data evaluating the therapeutic role of antithrombin III or immunomodulatory drugs [9–12]. However, the data from these RCTs may be of limited use to make epidemiological inferences because these studies represent a highly selective population group. There are also inconsistencies among the various studies evaluating the risk factors for development of thrombocytopenia [5, 7]. A recent study evaluating the incidence and risk factors for thrombocytopenia in septic shock patients identified a higher SOFA score (mean SOFA score of 12.7) as an independent risk factor for the development of thrombocytopenia [8]. This finding has limited utility because one of the factors used to calculate the SOFA score is actually the platelet count.

The objective of this study was to evaluate the incidence, risk factors, and prognostic importance of thrombocytopenia in patients admitted to an ICU with severe sepsis or septic shock. We also investigated the relationship between thrombocytopenia and major bleeding episodes and transfusion requirements.

## Methods

This is a retrospective cohort study conducted at the Mayo Clinic Medical Center, Rochester, MN. The study was approved by the Institutional Review Board. Informed consent was waived because of the anonymous and observational nature of this study. Adult patients (age ≥ 18 years) admitted to the 24-bed medical ICU at the St. Marys Hospital, Rochester, MN with a diagnosis of severe sepsis or septic shock between December 2007 and January 2009 were included in the study. Study patients were identified from the existing Sepsis Quality Initiative database, which contains data of patients admitted to the medical ICU with sepsis at the Mayo Medical Center. Only the first admission was included for patients who had multiple ICU admissions. Patients who have not authorized their medical records to be reviewed for research were excluded. Other exclusion criteria were history of platelet disorders (e.g., idiopathic thrombocytopenia purpura, congenital thrombocytopenia, hypersplenism), hematologic malignancies, use of chemotherapy (in the last 30 days prior to admission), mechanical heart valves, alcohol abuse, and hepatic cirrhosis.

The following data were abstracted from the patients’ electronic medical records: age, ethnicity, gender, co-morbidities, stage of sepsis, compliance with institutional sepsis resuscitation bundle, source of infection, microbial organism, development of acute lung injury (ALI) or acute respiratory distress syndrome (ARDS), duration of mechanical ventilation, vasopressor use, major bleeding episodes, transfusion requirements, duration of ICU stay, and 28-day mortality. The APACHE III score and predicted mortality were abstracted from the ICU Data Mart of our institution [13]. For patients who developed acute kidney injury, worst risk of renal dysfunction, injury to the kidney, failure of kidney function, loss of kidney function, and end-stage kidney disease (RIFLE) stage was determined [14]. The following laboratory values were collected for all patients when available: hemoglobin, creatinine, lactate, albumin, daily platelet count (up to 14 days or death whichever happened earlier), prothrombin time, activated partial thromboplastin time, D-dimer assay, fibrinogen level, peripheral blood smear evidence of hemolysis, serum lactate dehydrogenase, serum hepatic function panel, haptoglobin, and anti-platelet factor 4-heparin antibody by enzyme-linked immunosorbent assay (ELISA). Data about the usage of medications that are commonly associated with thrombocytopenia in critically ill patients was recorded [15]. We reviewed whether the study patients received beta-lactam antibiotics, vancomycin, linezolid, trimethoprim-sulfamethoxazole, H_2_ receptor antagonists, or heparin products (unfractionated and low-molecular weight heparin). One-time orders and medications prescribed ‘as needed’ were excluded.

### Definitions

Thrombocytopenia was defined as having a platelet count of <150 × 10^3^/μL (based on the hospital laboratory’s reference range and from published literature) [8, 16]. If more than one platelet value is available for a day, the lowest value was used. Severity of thrombocytopenia was classified into the following categories on the basis of nadir platelet count [8]: mild 101–149 × 10^3^/μL, moderate 51–100 × 10^3^/μL, severe 21–50 × 10^3^/μL, and very severe ≤20 × 10^3^/μL. Severe sepsis and septic shock were defined according to published guidelines [17]. Compliance with the institutional sepsis resuscitation bundle was met if the patient received evidence-based sepsis resuscitation care within 6 h as per the Surviving Sepsis Campaign guidelines [18]. Sites of infection were defined as per the guidelines from the International Sepsis Forum Consensus Conference [19]. ALI and ARDS were identified as per the American-European Consensus Conference definition [20]. Overt DIC was defined according to the International Society on Thrombosis and Haemostasis (ISTH) scoring system [21]. In case of missing data, normal values were assumed for calculation of the DIC score. Immune-mediated, heparin-induced thrombocytopenia (HIT) was defined as the presence of clinical criteria suggestive of HIT and the presence of platelet factor-4 antibodies [22]. Drug-induced thrombocytopenia was defined according to the published criteria as definite, probable, possible, or unlikely [23]. Major bleeding was defined as any intracranial or retroperitoneal bleed or overt bleeding (visible or symptomatic bleeding) with a decrease of hemoglobin concentration by more than 2 g/dL (20 g/L), or the requirement for transfusion of two or more units of packed red blood cells [24]. Multiple bleeding events from the same bleeding site/source were counted only once for each patient.

Definitions for co-morbidities: Hypertension was identified if patient had a known diagnosis of hypertension or was using anti-hypertensive medications. Diabetes mellitus was identified if the patient’s medical record contained an established diagnosis and use of any of the following: oral hypoglycemic drugs, insulin, or exanetide. Chronic lung disease was known to be present if patient’s medical record had an established diagnosis of asthma, chronic obstructive pulmonary disease, cystic fibrosis or interstitial lung disease. Coronary artery disease and congestive heart failure were identified if the medical record documented an established diagnosis of same. Alcohol abuse was defined as a known diagnosis of chronic alcoholism, a previous admission for alcohol detoxification or alcohol withdrawal, or reported alcohol consumption of more than two drinks per day or >14 drinks/week [25].

### Statistical analysis

Data were summarized as mean (standard deviation (SD)), median (interquartile range (IQR)) or percentages. We used unpaired Student’s *t* test to compare continuous variables with normal distribution and Mann-Whitney *U* test for skewed distribution. For comparison of categorical variables, we used chi-square test if the number of elements in each cell were 5 or higher and Fisher’s exact test, otherwise. Multivariate logistic regression analysis was performed to determine independent risk factors associated with the development of thrombocytopenia. Univariate analysis was done to identify the candidate variables for multiple logistic regression analysis. Colinear variable with a low *P* value (≤0.1) or variables that were thought to have strong biologic associations were included in the model. To determine the impact of thrombocytopenia on mortality, we performed a multivariate logistic regression analysis using the 28-day mortality as the dependent factor. Variables considered for the multivariate modeling included age, APACHE III score, and compliance with sepsis resuscitation bundle. The performance of the multiple logistic regression models were assessed by the area under the receiver operating characteristic curve statistics. When appropriate, the odds ratio (OR) and 95% confidence intervals (CI) were calculated. A *P* value of <0.05 was considered statistically significant. The JMP statistical software (version 8.0; SAS Institute, Inc, Cary, NC, USA) was used for all data analyses.

## Results and discussion

### Results

Between December 28, 2007 and January 12, 2009, 387 patients were admitted to the ICU with severe sepsis or septic shock, of whom 83 patients were excluded from the study due to preexisting exclusion criteria. The reasons for exclusion included lack of research authorization (14 patients), cirrhosis (20 patients), hematologic malignancy (17 patients), alcoholism (15 patients), chronic thrombocytopenic disorder (7 patients), active chemotherapy (6 patients), and a mechanical heart valve (4 patients). A total of 304 patients were included in the study, of whom 171 (56.2%) were male patients. The patients’ mean (±SD) age was 68.8 (±15.8) years and majority of them were Caucasians (269 out of 304, 88.4%). Septic shock was present in 285 out of 304 patients (93.7%), and the most common source of sepsis was pneumonia (Table 1).

**Table 1:** **Source of sepsis for all patients admitted to ICU (** ***n*** = **304)**

Source of sepsis	Number of sources (%)
Pneumonia	118 (38.8)
Urinary tract	57 (18.8)
Intra-abdominal	29 (9.5)
Skin and soft tissue infection	18 (5.9)
Blood stream infection	13 (4.3)
Other sources	20 (6.6)
Unknown	49 (16.1)

ICU, intensive care unit.

Thrombocytopenia developed in 145 patients (47.6%); 77 (25.3%) patients had low platelet count at the time of ICU admission and an additional 68 (22.3%) patients developed thrombocytopenia during their hospital course. The median (IQR) duration of thrombocytopenia was 4.4 (1.9–6.9) days. There were no significant differences in the stage of sepsis and the compliance with sepsis resuscitation bundle for patients with thrombocytopenia as compared to patients who did not develop thrombocytopenia. Patients with thrombocytopenia had higher APACHE III scores compared to patients who did not have thrombocytopenia (*P* < 0.01) (Table 2).

**Table 2:** **Demographics and clinical characteristics of 304 patients admitted to ICU with sepsis**

Variable	Thrombocytopenia	No thrombocytopenia	***P*** value
	( ***n*** = 145)	( ***n*** = 159)	
Age (years), mean (SD)	69.5 (15.4)	68.2 (16.1)	0.55
Male gender, *n* (%)	85 (58.6)	86 (54.1)	0.42
White race, *n* (%)	129 (88.9)	140 (88.1)	0.8
Hypertension, *n* (%)	108 (74.4)	120 (75.4)	0.84
Diabetes, *n* (%)	55 (37.9)	55 (34.5)	0.54
Chronic lung disease, *n* (%)	36 (24.8)	56 (35.2)	0.04*
Chronic kidney disease, *n* (%)	46 (31.7)	36 (22.6)	0.07
Coronary artery disease, *n* (%)	46 (31.7)	63 (39.6)	0.15
Congestive heart failure, *n* (%)	35 (24.1)	43 (27)	0.56
Septic shock, *n* (%)	135 (93.1)	150 (94.3)	0.65
Compliance with sepsis bundle, *n* (%)	62 (42.7)	78 (49.1)	0.27
APACHE III score, mean (SD)	81.7 (29.7)	71.5 (26.4)	0.001*
Age adjusted Charlson Index, median (IQR)	5 (3–9)	6 (3–8)	0.5

ICU, intensive care unit; SD, standard deviation; APACHE, Acute Physiology, Age, and Chronic Health Evaluation; IQR, interquartile range. *Significant *P* values.

Patients with thrombocytopenia had significantly elevated serum lactic acid levels at ICU admission (median value, 2.7 mmol/L vs. 1.8 mmol/L, *P* < 0.01). Patients with thrombocytopenia also had elevated serum creatinine and bilirubin levels and lower serum albumin compared to the patients without thrombocytopenia (Table 3). There were no statistically significant differences in serum hemoglobin, prothrombin time, partial thromboplastin time, and hepatic transaminase levels between these two groups.

**Table 3:** **Laboratory values at admission to the intensive care unit**

Variable	Thrombocytopenia	No thrombocytopenia	***P*** value
	( ***n*** = 145)	( ***n*** = 159)	
Hemoglobin (g/dL), mean (SD)	11.3 (2.1)	11.3 (2.2)	0.81
Albumin (g/dL), mean (SD)	2.5 (0.6)	2.8 (0.4)	0.015*
(*n* = 132)	(*n* = 75)	(*n* = 57)	
Lactate (mmol/L), median (IQR)	2.7 (1.4–4.1)	1.8 (1.1–2.7)	<0.0001*
(*n* = 299)	(*n* = 142)	(*n* = 157)	
Creatinine (mg/dL), median (IQR)	1.6 (1.1–3.2)	1.3 (0.8–2.2)	0.002*
Total Bilirubin (mg/dL), median (IQR)	0.7 (0.4–1.3)	0.5 (0.3-0.5)	0.001*
(*n* = 216)	(*n* = 116)	(*n* = 100)	
Day 1 net fluid balance (L), median (IQR)	5.1 (2.9–8.0)	4.7 (2.4-7.1)	0.16
Day 2 net fluid balance (L), median (IQR)	1.8 (0.2–3.4)	1.6 (0.2–3.4)	0.59
(*n* = 298)	(*n* = 143)	(*n* = 155)	

SD, standard deviation; IQR interquartile range. SI conversion factors: to convert hemoglobin and albumin values to g/L, multiply by 10; to convert creatinine values to μmol/L, multiply by 88.4; to convert total bilirubin values to μmol/L, multiply by 17.104. *Significant *P* values.

Of the 145 patients who developed thrombocytopenia, 37 (25.5%) were found to have overt DIC. Possible drug-induced thrombocytopenia developed in 26 of 145 patients (17.9%), and all the cases were thought to be secondary to antibiotics. ELISA to detect heparin-platelet factor 4 antibodies was performed in 49 patients. Two patients had equivocal test results but later ruled out and did not have immune-mediated HIT. One patient had a positive test result and was diagnosed with immune-mediated HIT (Table 4).

**Table 4:** **Clinical data in 145 patients who developed thrombocytopenia**

Variable	Value
Development of thrombocytopenia	
At the time of ICU admit, *n* (%)	77 (25.3)
During the ICU/hospital course, *n* (%)	68 (22.3)
Duration of thrombocytopenia (days), median (IQR)	4.4 (1.9–6.9)
Resolution of thrombocytopenia by hospital discharge, *n* (%)	86 (59.3)
Severity of thrombocytopenia	
Mild, *n* (%)	72 (49.6)
Moderate, *n* (%)	47 (32.4)
Severe, *n* (%)	19 (13.1)
Very severe, *n* (%)	7 (4.8)
Disseminated intravascular coagulation, *n* (%)	37 (25.5)
Drug-induced thrombocytopenia, *n* (%)	26 (17.9)
Heparin-induced thrombocytopenia, *n* (%)	1 (0.6)

ICU, intensive care unit; IQR, interquartile range.

Patients who developed thrombocytopenia had more episodes of major bleeding compared to those patients who did not develop thrombocytopenia (14.4% vs. 3.7%, *P* < 0.01). Patients with thrombocytopenia also received more blood product transfusions. Sixty-four of 145 patients with thrombocytopenia developed acute kidney injury (worst RIFLE stage of ‘injury’ or more) compared to 47 of 159 patients without thrombocytopenia (*P* < 0.01). Patients with thrombocytopenia received prolonged vasopressor support and had longer ICU lengths of stay. ICU mortality and 28-day mortality were not significantly different between these two groups (Table 5). Patients with higher severity of thrombocytopenia had higher incidence of ALI/ARDS. Twenty-three out of 73 patients (31.5%) with moderate or higher severity of thrombocytopenia developed ALI or ARDS compared to 39 out of 231 patients (16.8%) with no or mild thrombocytopenia (*P* < 0.01). Although the mortality increased with severity of thrombocytopenia, it did not reach a statistical significance. The 28-day mortality was 25.5% (59 out of 231) in patients with no or mild thrombocytopenia, compared to 37% (27 out of 73) in patients with the thrombocytopenia severity of moderate or higher (*P* = 0.06).

**Table 5:** **Comparison of clinical outcome data for patients with and without thrombocytopenia**

Variable	Thrombocytopenia	No thrombocytopenia	***P*** value
	( ***n*** = 145)	( ***n*** = 159)	
Major bleeding, *n* (%)	21 (14.4)	6 (3.7)	0.001*
PRBC transfusion, *n* (%)	85 (58.6)	62 (38.9)	0.0006*
FFP transfusion, *n* (%)	24 (16.5)	15 (9.4)	0.06
Platelet transfusion, *n* (%)	16 (11.3)	0	<0.0001*
Cryoprecipitate transfusion, *n* (%)	8 (5.5)	0	0.002*
Acute kidney injury (worst RIFLE stage ‘injury’ or more), *n* (%)	64 (44.1)	47 (29.5)	0.008*
Vasopressor duration (h), median (IQR)	37 (17–76)	23 (13–46)	0.004*
ALI/ARDS, *n* (%)	33 (22.8)	29 (18.2)	0.32
Mechanical ventilation, *n* (%)	75 (51.7)	78 (49.1)	0.64
Mechanical ventilation duration (days), median (IQR)	5.1 (2.1–9.2)	3 (1.1–9.2)	0.11
ICU length of stay (days), median (IQR)	3.1 (1.6–7.8)	2.1 (1.2–4.4)	0.001*
ICU mortality, *n* (%)	24 (16.5)	19 (11.9)	0.25
28-day mortality, *n* (%)	47 (32.4)	39 (24.5)	0.12

PRBC, packed red blood cells; FFP, fresh frozen plasma; RIFLE, risk of renal dysfunction, injury to the kidney, failure of kidney function, loss of kidney function and end-stage kidney disease; IQR, interquartile range; ALI, acute lung injury; ARDS, acute respiratory distress syndrome; ICU, intensive care unit. * Significant *P* values.

The risk factors associated with development of thrombocytopenia according to multivariate logistic regression analysis are shown in Table 6. Candidate variables with significant *P* values in univariate analysis were entered into the model. Elevated serum lactate and prolonged vasopressor duration were independent risk factors for development of thrombocytopenia, after adjusting for age and compliance with the institutional sepsis resuscitation bundle.

**Table 6:** **Multivariate logistic regression model analyzing various risk factors for development of thrombocytopenia in 304 patients**

Variable	Odds ratio	95% confidence interval	***P*** value
APACHE III score	0.99	0.98–1.02	0.9
Lactate	1.59	1.1–2.47	0.02*
Albumin	0.66	0.25–1.74	0.4
Creatinine	1.07	0.78–1.51	0.6
Vasopressor duration	1.02	1.00–1.03	0.02*
Acute kidney injury (RIFLE stage ‘injury’ or more)	1.43	0.47–4.31	0.5

ICU, intensive care unit; APACHE, Acute Physiology, Age, and Chronic Health Evaluation; RIFLE, risk of renal dysfunction, injury to the kidney, failure of kidney function, loss of kidney function and end-stage kidney disease. *Significant *P* values.

**Figure 1:**
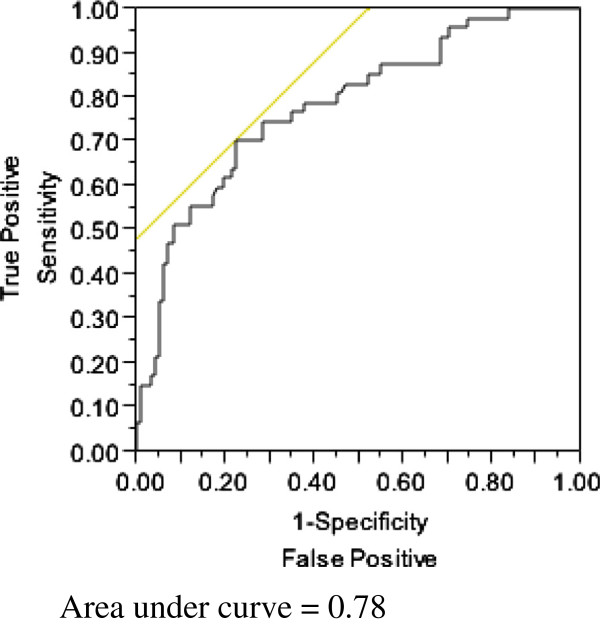
**Receiver-operator characteristic curve analysis showing non-resolution of thrombocytopenia as a predictor of 28-day mortality.**

Eighty-six of 145 patients who developed thrombocytopenia had normal platelet count values by the time of discharge from the hospital. Patients with persistent thrombocytopenia had higher ICU, hospital, and 28-day mortality values compared to those patients whose platelet counts normalized during hospitalization (Table 7). Fifteen of 86 patients (17.4%) who resolved their thrombocytopenia by the time of discharge from hospital died, compared to 32 of 59 (54.2%) who did not resolve their thrombocytopenia (*P* < 0.01). The association between non-resolution of thrombocytopenia and 28-day mortality remained significant after adjusting for age, APACHE III score, and compliance with sepsis resuscitation bundle (*P* < 0.01), with a receiver operating characteristic (ROC) area under curve (AUC) of 0.78 (Figure 1).

**Table 7:** **Mortality data among 145 patients with thrombocytopenia**

Variable	Thrombocytopenia resolved by hospital discharge	Thrombocytopenia not resolved by hospital discharge	***P*** value
	( ***N*** = 86)	( ***N*** = 59)	
ICU mortality, *n* (%)	4 (4.6)	20 (33.9)	<0.0001*
Hospital mortality, *n* (%)	15 (17.4)	24 (40.6)	0.002*
28-day mortality, *n* (%)	15 (17.4)	32 (54.2)	<0.0001*

Stratified according to the resolution of thrombocytopenia by the time of hospital discharge. ICU, intensive care unit. *Significant *P* values.

## Discussion

In this retrospective study of 304 patients admitted to ICU with severe sepsis or septic shock, thrombocytopenia developed in 145 (47.6%) patients. The majority of the patients (285 out of 304 patients, 93.7%) had septic shock, and the most common source of sepsis was pneumonia. Patients with thrombocytopenia had higher severity of illness scores (APACHE III) and elevated lactate levels compared to the patients who did not have thrombocytopenia. Elevated serum lactate and prolonged vasopressor duration were found to be independent risk factors for development of thrombocytopenia after adjusting for age and compliance with the institutional sepsis resuscitation bundle. Patients with thrombocytopenia were more likely to develop major bleeding episodes and to receive more blood product transfusions. Patients who developed thrombocytopenia had a higher incidence of acute kidney injury, required prolonged vasopressor support, and had a prolonged ICU stay. ICU mortality and 28-day mortality were not significantly different between these two groups. Although mortality increased with the severity of thrombocytopenia, the association between these two variables did not reach a statistical significance. Of the 145 patients who developed thrombocytopenia, 86 (59.3%) had normal platelet counts by the time of discharge from the hospital. Patients who did not resolve their thrombocytopenia by the time of hospital discharge had higher mortality compared to those patients whose platelet counts were normalized by the time of hospital discharge. The association between non-resolution of thrombocytopenia and 28-day mortality remained significant after adjusting for age, APACHE III score, and compliance with sepsis resuscitation bundle.

The incidence of thrombocytopenia was 47.6% in our study patients. Previous studies that specifically evaluated thrombocytopenia in patients with infection reported an incidence of 22%–58% [8, 26–29]. This wide variation in incidence of thrombocytopenia is likely explained by the disparate characteristics of these study patients, as well as differences in how thrombocytopenia was defined. Gafter-Gvili et al. reported an incidence of thrombocytopenia of 22.3% in patients with *Staphylococcus aureus* bacteremia; however, only 11% of their patients had septic shock [26]. In our study, 94% of patients were diagnosed with septic shock. Other studies that included more severely ill patients reported an incidence of thrombocytopenia comparable to our findings. In a study of patients with septic shock, 55% developed thrombocytopenia [8], and another study evaluating thrombocytopenia in ICU patients with nosocomial blood stream infections reported an incidence of 43% [29]. When thrombocytopenia was defined as a platelet count of <100 × 10^3^/μL, the incidence was reported to be about 33%–36% [28, 29]. We defined thrombocytopenia as platelet count <150 × 10^3^/μL, which could explain the higher incidence of thrombocytopenia in our study.

The development of thrombocytopenia in patients with sepsis is secondary to various mechanisms. Platelets are activated in sepsis and bound to the endothelium, resulting in sequestration and destruction [30, 31]. Immune-mediated mechanisms like nonspecific platelet-associated antibodies [32] and cytokine-driven hemophagocytosis of platelets [33, 34] can also contribute to sepsis-induced thrombocytopenia. Severe sepsis is commonly associated with a net procoagulant state, and secondary consumptive conditions of thrombocytopenia, like DIC, represent an extreme in the continuum of hemostatic abnormalities in patients with sepsis. In our study, 25% of patients with thrombocytopenia had overt DIC. This number is similar to earlier reports which estimated that 15%–30% of patients with severe sepsis and septic shock develop DIC [35]. Although DIC is considered to be a predictor of mortality, we did not find an association between the presence of overt DIC and mortality in our study. Immune-mediated HIT was diagnosed in only one patient in our study cohort. Although sepsis is not a known risk factor for TTP, recent reports have suggested that infections can trigger TTP in patients with preexisting risk factors [36, 37]. We did not find any case of TTP in our study.

Many studies tried to identify potential risk factors for the development of thrombocytopenia in the ICU. Sepsis was found to be the most common risk factor in several studies [2, 5–7]. Increased severity of illness (as suggested by high APACHE II and SOFA scores) [2, 8], presence of invasive vascular catheters (e.g., central venous catheter, pulmonary artery catheter) [6, 7] and drugs (heparin, beta-lactam antibiotics, and vancomycin) [5, 6] were also suggested to be risk factors for the development of thrombocytopenia; however, these findings have not been consistent among different studies. We found that elevated serum lactate and prolonged vasopressor duration were independent risk factors for thrombocytopenia. It is plausible that these risk factors reflect the severity of underlying septic process and critical illness rather than predicting the causality. We did not specifically evaluate for effects of specific drugs on the development of thrombocytopenia in our patient cohort.

One of the main concerns for critically ill patients with thrombocytopenia is bleeding. We found that patients with thrombocytopenia were more likely to develop major bleeding episodes and they also received more blood product transfusions. Blood product transfusion is a risk factor for the development of lung injury including transfusion-associated acute lung injury (TRALI). Patients with higher severity of thrombocytopenia had more major bleeding episodes and they also had a higher incidence of ALI or ARDS. We found that patients with thrombocytopenia had a higher incidence of acute kidney injury and required vasopressor support for longer duration compared to the patients without thrombocytopenia. This may explain the observed longer ICU length of stay for patients with thrombocytopenia in our study.

Thrombocytopenia is a known negative prognostic indicator for adverse clinical outcomes in ICU patients. Previous studies have reported that thrombocytopenia carries an independent risk for mortality in ICU patients [3, 4, 38]. However, in our study, we did not find a significant difference in the ICU mortality or 28-day mortality between the patients with and without thrombocytopenia. This could be due to differences in the study population, as our study included only patients with severe sepsis/septic shock, whereas previous studies included critically ill patients from both medical and surgical ICUs. In another study of 145 critically patients from a medical ICU, Strauss and colleagues also found that thrombocytopenia alone was not a predictor of mortality. A drop in platelet count of ≥30%, not thrombocytopenia *per se* predicted ICU mortality in their study [16]. Our study results also differ from that of a recent study by Sharma et al., who reported that thrombocytopenia is a predictor of mortality in patients with septic shock [8]. Although they found that the unadjusted 6-month mortality rate was higher in patients with thrombocytopenia, the receiver operator characteristic curve analysis showed that thrombocytopenia has poor sensitivity as a marker for predicting mortality. The hazard ratios for estimating the increased probability of mortality for patients with thrombocytopenia also had wide confidence intervals in their study, thereby minimizing the effect size. Brogly and colleagues reported that a platelet count of <150 × 10^9^/L did not predict mortality in patients admitted to ICU for severe community-acquired pneumonia [39]. However, the ICU mortality has increased significantly with severity of thrombocytopenia in their study, with an initial platelet count ≤50 × 10^9^/L independently predicting the ICU mortality. It is plausible that severity of thrombocytopenia could be a better predictor of mortality rather than mere presence of thrombocytopenia.

We found that non-resolution of thrombocytopenia during the recovery from critical illness is a strong predictor of mortality. Patients with persistent thrombocytopenia had higher ICU, hospital, and 28-day mortality compared to those patients whose platelet counts have normalized. This association remained significant after adjusting for age, APACHE III score, and compliance with sepsis resuscitation bundle. An acute decrease in platelet counts secondary to a major inflammatory event is commonly followed by thrombocytosis reflecting bone marrow activation. Lack of this biphasic response of platelet recovery portends poor outcomes. Akca and colleagues [4] found that a prolonged thrombocytopenia and an absence of relative increase (a 25% increase above the admission value) in platelet count were associated with mortality in critically ill patients. The absence of relative increase in the platelet count on day 14 was associated with mortality even in patients without thrombocytopenia. Similar findings were reported by Nijsten and colleagues in a group of surgical ICU patients [40]. Our results support the findings from the abovementioned studies that have shown that changes in platelet counts over time may have greater prognostic significance rather than the absolute counts in critically ill patients. Our results suggest that even in patients who survived the acute illness from severe sepsis/septic shock and discharged from ICU, lack of resolution of thrombocytopenia portends a bad prognosis.

Care of sepsis patients has improved significantly in the last decade with the implementation of sepsis resuscitation ‘bundles’, which led to improved clinical outcomes. It is unknown whether the prognostic significance of thrombocytopenia has changed with the advances in the care of sepsis patients in the ICU. We have included compliance with sepsis resuscitation bundle as one of the variables in our statistical analyses, and adjusted its effect on determining the association between thrombocytopenia and mortality. The association between non-resolution of thrombocytopenia and 28-day mortality remained significant even after adjusting for the compliance with sepsis resuscitation bundle. This finding is significant because lack of resolution of thrombocytopenia still holds an important prognostic value despite recent advances in sepsis care.

Our study has several limitations. Since it is a retrospective study, we could not control for all confounding factors that could have been present in the critically ill patients at different times. Compliance with a sepsis resuscitation bundle is an important factor that affects mortality in patients with severe sepsis or septic shock. Previous studies evaluating thrombocytopenia in septic shock patients [8] did not adjust for this important confounding factor. Our study results were adjusted for compliance with sepsis resuscitation bundle, which strengthens the study findings. Although we included 304 patients in our study, mortality rate in our study is relatively low compared to other studies. Because of the relatively low number of deaths, our analyses are not adequately powered to determine statistically significant differences between groups. Our sample size was also not sufficient enough to analyze the incidence or impact of rare events like heparin-induced thrombocytopenia, etc.

## Conclusions

In conclusion, this is the largest study evaluating the incidence and clinical outcomes of thrombocytopenia in patients admitted to the ICU with severe sepsis and septic shock. Thrombocytopenia is common among patients with sepsis admitted to the ICU and has prognostic importance. Patients with thrombocytopenia tend to be sicker and are more likely to develop major bleeding episodes. Although our results did not find an association between presence of thrombocytopenia and 28-day mortality, patients with thrombocytopenia had significant morbidity as evidenced by higher incidence of acute kidney injury and longer ICU stay, compared to the patients who did not develop thrombocytopenia. Among patients who developed thrombocytopenia, non-resolution of thrombocytopenia was associated with increased mortality. The association between non-resolution of thrombocytopenia and 28-day mortality remained significant after adjusting for age, APACHE III score, and compliance with sepsis resuscitation bundle.

## Authors’ information

BA passed away on January 10, 2013.

Christopher Farmer is a Professor of Medicine at the Mayo Clinic College of Medicine. He is the Chair of the Department of Critical Care Medicine at Mayo Clinic, Arizona. He is also the President-elect of the Society of the Critical Care Medicine.

## Abbreviations

ALI: Acute lung injury
APACHE: Acute physiology, Age, and Chronic Health Evaluation
ARDS: Acute respiratory distress syndrome
DIC: Disseminated intravascular coagulation
ELISA: Enzyme-linked immunosorbent assay
FFP: Fresh frozen plasma
HIT: Heparin-induced thrombocytopenia
ICU: Intensive care unit
IQR: Interquartile range
PRBC: Packed red blood cells
RIFLE: Risk of renal dysfunction, injury to the kidney, failure of kidney function, loss of kidney function and end-stage kidney disease
ROC: Receiver operating characteristic
SD: Standard deviation
SOFA: Sequential organ failure assessment
TRALI: Transfusion-associated acute lung injury
TTP: Thrombotic thrombocytopenic purpura.
